# De Winter T waves: an ominous sign of ST-elevation myocardial infarction

**DOI:** 10.11604/pamj.2022.42.168.35910

**Published:** 2022-07-01

**Authors:** Sze Shian Wee, Kian Seng Ng

**Affiliations:** 1 Medicare, 118 Jalan Mersing, Kluang, Johor, Malaysia

**Keywords:** De winter T waves, ST elevation, myocardial infarction, descending artery occlusion

## Image in medicine

A 46-year-old gentleman presented with sudden retrosternal gripping pain. Creatinine Kinase was >6000 units/L. Patient was diagnosed as Non-ST Elevation Myocardial Infarction (NSTEMI). The presenting electrocardiogram (ECG) was shared with us (A). Our response was, “This is not a NSTEMI. ECG manifests de Winter T Waves. The de Winter complex is an upsloping J-point ST depression (STD) that continues into a tall, positive symmetrical T Wave. Do not be misled by the STD. This is a STEMI without the signature ST-segment elevation (STE). De Winter T Waves is a STEMI equivalent; it signifies acute occlusion of the proximal left anterior descending artery (LADA).” De Winter T Waves was first reported by in 2008. Unfortunately, knowledge of de Winter T Waves is not widespread and it is often misdiagnosed as NSTEMI. Failure to carry out emergent reperfusion will negatively impact morbidity and mortality. ECG in (A) manifests the following features which points to de Winter T Waves: (1) de Winter T Waves occur in a pain state; (2) ST segment showed a 1-3 mm upsloping STD at the J point in precordial leads; this is best exemplified by V4; (3) upsloping STD continues into tall, positive symmetrical T Waves; (2) and (3) are seen in (C) part of image; (4) ST segment elevation (STE) in aVR, V1; (5) loss of anterior R waves (V1 to V4) and (6) reciprocal STD seen in leads II, III, aVF. de Winter T Waves is a unique ECG sign. Do not be deceived by the STDs into diagnosing NSTEMI. It is a STEMI pointing to acute occlusion of the LAD.

**Figure 1:**
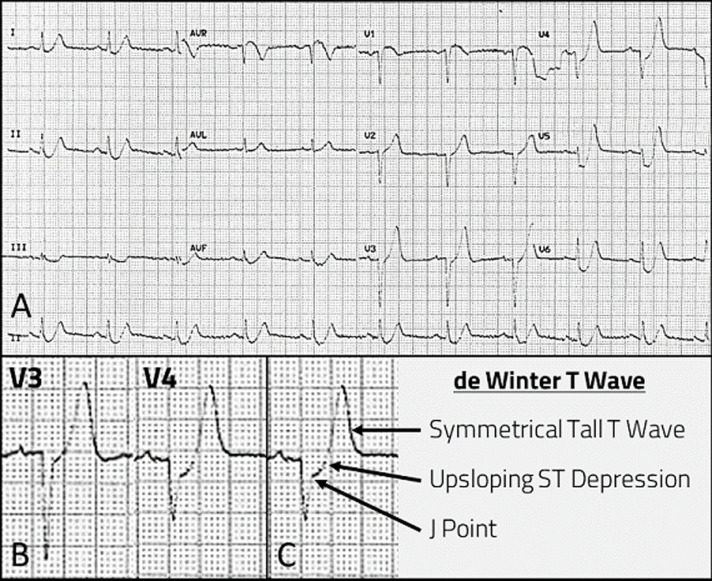
A) the 12 lead ECG taken at time of ongoing chest pain; B) leads V3, V4 enlarged; C) de Winter T Wave complex illustrated

